# Depth-dependence and monthly variability of charophyte biomass production: consequences for the precipitation of calcium carbonate in a shallow *Chara*-lake

**DOI:** 10.1007/s11356-016-7420-8

**Published:** 2016-08-22

**Authors:** Andrzej Pukacz, Mariusz Pełechaty, Marcin Frankowski

**Affiliations:** 1 Polish-German Research Institute, Collegium Polonicum, Kościuszki 1, 69-100 Słubice, Poland; 2 Department of Hydrobiology, Faculty of Biology, Adam Mickiewicz University in Poznań, Umultowska 89, 61-614 Poznań, Poland; 3 Department of Water and Soil Analysis, Faculty of Chemistry, Adam Mickiewicz University in Poznań, Umultowska 89b, 61-614 Poznań, Poland

**Keywords:** Charophytes, Biomass production, Calcium carbonate precipitation, Temporal variability, Nutrient cycling

## Abstract

The month-to-month variability of biomass and CaCO_3_ precipitation by dense charophyte beds was studied in a shallow *Chara*-lake at two depths, 1 and 3 m. Charophyte dry weights (d.w.), the percentage contribution of calcium carbonate to the dry weight and the precipitation of CaCO_3_ per 1 m^2^ were analysed from May to October 2011. Physical-chemical parameters of water were also measured for the same sample locations. The mean dry weight and calcium carbonate precipitation were significantly higher at 1 m than at 3 m. The highest measured charophyte dry weight (exceeding 2000 g m^−2^) was noted at 1 m depth in September, and the highest CaCO_3_ content in the d.w. (exceeding 80 % of d.w.) was observed at 3 m depth in August. The highest CaCO_3_ precipitation per 1 m^2^ exceeded 1695 g at 1 m depth in August. Significant differences in photosynthetically active radiation (PAR) were found between 1 and 3 m depths; there were no significant differences between depths for other water properties. At both sampling depths, there were distinct correlations between the d.w., CaCO_3_ content and precipitation and water properties. In addition to PAR, the water temperature and magnesium and calcium ion concentrations were among the most significant determinants of CaCO_3_ content and d.w. The results show that light availability seems to be the major factor in determining charophyte biomass in a typical, undisturbed *Chara*-lake. The study results are discussed in light of the role of charophyte vegetation in whole ecosystem functioning, with a particular focus on sedimentary processes and the biogeochemical cycle within the littoral zone.

## Introduction

Primary production in freshwater ecosystems is regulated by the photosynthetic activity of aquatic macrophytes in the littoral zone and by phytoplankton in the pelagic zone (Yates and Robbins 1998; Dodds 2002; Dittrich et al. 2004). In shallow, macrophytic lakes, plants covering most of the lake basin play the leading role in the biomass production. In contrast, phytoplanktons are responsible for the majority of photosynthetic productivity in deep, stratified lakes (Alimov 2003). Among aquatic macrophytes, charophytes (stoneworts), a green macroscopic algae belonging to the family *Characeae*, are regarded as a special group. Charophytes can be found in almost all types of aquatic ecosystems (Forsberg 1965; Krause 1997; Urbaniak and Gąbka 2014). However, the occurrence of most charophytes is limited to water bodies with clear, alkaline waters and low rates of nutrient input to the system (Hutchinson 1975; Murphy et al. 1983; McConnaughey 1997; Krause 1997). Hence, charophytes become rare with increasing trophic level and decreasing light availability, and many species are used as sensitive bioindicators of water quality and habitat conditions (e.g. Blindow 1992a; Menendez and Sanchez 1998; Schwarz et al. 1999; Kłosowski et al. 2006).

Due to the multi-faceted role of charophytes, they are currently considered to be an important habitat-forming factor of aquatic ecosystems (Rodrigo et al. 2007; Pukacz et al. 2013). This group of hydrophytes plays a significant role in freshwater food webs by providing a refuge for invertebrates (e.g. Van den Berg et al. 1998; Van Donk and van de Bund 2002) and serving as a food resource for fish and birds (e.g. Schmeider et al. 2006; Pípalová 2006; Dibble and Kovalenko 2009). Charophytes are also competitors for nutrients with phytoplankton and periphyton (Forsberg et al. 1990; Van Donk and van de Bund 2002). By building dense meadows, charophytes also increase sedimentation and reduce sediment re-suspension (Søndergaard et al. 1992; Van den Berg et al. 1998).

Probably the most significant way in which charophytes affect aquatic ecosystems is through biomass production, which is an important factor in nutrient cycling (Kufel and Kufel 2002). Charophytes may form dense meadows with biomass exceeding that of vascular plants (Blindow 1992b; Pełechaty et al. 2013). Hence, a significant portion of the nutrients within a system can be captured by these organisms (Rodrigo et al. 2007; Kufel et al. 2013). It is also noteworthy that a significant portion of charophyte dry mass may consist of carbonates (Pentecost 1984). This is due to their capacity to use bicarbonates during photosynthesis (Wetzel 2001), resulting in the deposition of insoluble calcium carbonate directly onto the surface of a charophyte thalli, forming a mineral encrustation (Raven et al. 1986; Martin et al. 2003). This process decalcifies the ambient water (influencing alkalinity and pH) and results in the storage of a large portion of incorporated carbon in the sediment (Rodrigo et al. 2009). Additionally, the deposition process is accompanied by the co-precipitation of inorganic phosphorus, enhancing its inactivation for a longer time (Murphy et al. 1983; Anderson and Ring 1999).

All the above effects stabilize the trophic status and stimulate the clear water state of a lake. Such an effect is often observed in shallow, hard water ecosystems, such as that examined herein. Although charophytes have a significant impact on lake ecosystems, there is still a paucity of published data on charophyte dry weight and associated carbonate participation. Most of the existing charophyte biomass and nutrient concentration data were measured at one time point. There is no data concerning the in situ variability of charophyte biomass production over a depth gradient. Hence, the goal of this study was to quantify the charophyte dry weight and carbonate precipitation over a whole vegetation season. We hypothesised that the depth would have a limiting effect on the production of dry weight and precipitation of carbonates due to decreasing light availability (measured herein as photosynthetically active radiation—PAR) and that this effect would vary over time. Additionally, we analysed which of the physical-chemical properties of ambient water affected charophyte dry weight and carbonate production.

## Materials and methods

### The study site

Material was collected from Lake Jasne, a small post-glacial lake located in the Lubuskie Lakeland, western Poland (52° 17′ 36″ N, 15° 02′ 11″ E). This is a shallow, tachymictic ecosystem, (area 15.1 ha, max. depth 9.5 m, mean depth 4.3 m), with no fully developed vertical stratification. Over 90 % of the drainage basin is forested (mostly by pine forests). It is one of the clearest mesotrophic lakes within the region, characterized by high water transparency and low nutrient concentrations (Pełechaty et al. 2015). In Lake Jasne, charophyte vegetation forms dense meadows, covering approximately 65 % of the lake bottom (Pukacz and Pełechaty 2013). The presence of stable, undisturbed vegetation was the main reason for choosing this lake for this study.

### Sampling and field measurements

The investigation was performed in the vegetation season of 2011. The field study started 1 month after ice melt (May) and finished when regular ground frosts began to appear (October). Each month, charophyte samples were taken for biomass analyses, and water samples were collected for physical-chemical analyses. Three sample sites (A, B and C) were selected with similar qualitative (domination of one species) and quantitative (100 % bottom coverage) community structure, as determined by previous observations. Each site was localized in the centre of a separate patch (30–50 m between patches) of *Chara polyacantha* A. Braun 1859, within the central shallows. All patches had a dense, uniform and monospecific structure. Plant samples were collected at two depths (1 and 3 m) along the bottom slope. At each site, samples of 0.04 m^2^ were cut manually using a steel frame (0.2 × 0.2 m) and a knife to minimize the edge effect. In consecutive months, new samples were taken ca. 50 cm from the previous one. The plant material was transported to the laboratory in plastic bags.

Prior to plant sampling, the basic physical-chemical parameters of water were measured, and samples were collected for further chemical analyses. Water samples were taken directly above the macrophyte beds at all sample sites and at each studied depth using an electric pump. Each month, all measurements and water sample collections were performed between 11:00 a.m. and 1:00 p.m. Dissolved oxygen concentration and temperature were measured using an Elmetron CX-401 portable metre, and electrolytic conductivity and pH were measured with a Cyber-Scan 200. PAR, expressed as a percentage of the subsurface value, was measured using a Li-Cor Spherical Quantum Sensor LI-193 connected to a Li-Cor 192 m. For chemical analyses, the samples were collected in 1-l plastic bottles and kept in a portable refrigerator. Alkalinity was determined in a laboratory within 6 h of sampling. Then, the samples were kept in a refrigerator (at 4 °C) until the remaining chemical analyses were performed.

### Laboratory analyses

Immediately after the field study, plant samples were air-dried for 24 h with laboratory ventilation to avoid decomposition. Plants were subsequently dried at 105 °C for 3 h in an electric drier in order to determine the dry plant weight (d.w.). Dry plant samples were analysed to determine the contents of organic matter, calcium carbonate and mineral residues. The calcium carbonate content (% CaCO_3_ in d.w.) was determined by the two-step weight loss on ignition method (Heiri et al. 2001). Powdered samples were first combusted at 550 °C for 4 h and subsequently at 950 °C for 2 h. Carbonate content was calculated by multiplying the mass of CO_2_ evolved in the second step of the analysis by 1.36. Finally, CaCO_3_ content was calculated by multiplying the CO_3_^2−^ content by 1.66. The loss on ignition at 550 °C is presumed to represent the percentage of organic matter.

Alkalinity was determined by titration method with indicator and colour with the visual method against the platinum scale. Total water hardness was determined by the versenate method. A Metrohm ion chromatograph (881 Compact IC Pro model, Metrohm, Switzerland) was used to determine Ca^2+^ and Mg^2+^, with Metrosep C4 Guard (with the 4.0 guard column) and Metrosep C4 150 (with the 4.0 separating column) columns. Total nitrogen was determined by a TOC-L Shimadzu analyser with a TNM-L unit using the catalytic thermal decomposition and chemiluminescence methods (Shimadzu, Japan). Total phosphorous was determined by ICP-OES 9820 (Shimadzu, Japan).

### Data analyses

Statistical analyses were performed using STATISTICA 10.1 software (StatSoft Inc., Tulsa, OK, USA). The normality of the distributions of the analysed variables and the homoscedasticity of the samples were tested with the Shapiro-Wilk and the Levene tests, respectively. If both conditions were satisfied, a one-way ANOVA and Fisher’s least significant difference post hoc test were used to compare the means of the variables. Otherwise, non-parametric tests were used. The relationships between the mean values measured at different sampling times and depths were analysed with a two-way ANOVA and post hoc Scheffé test. Because the number of samples was limited, a Spearman rank correlation was applied to test the relationships between charophyte dry mass, calcium carbonate content and the physical-chemical properties of water.

## Results

### The dry mass variability

**Fig. 1:**
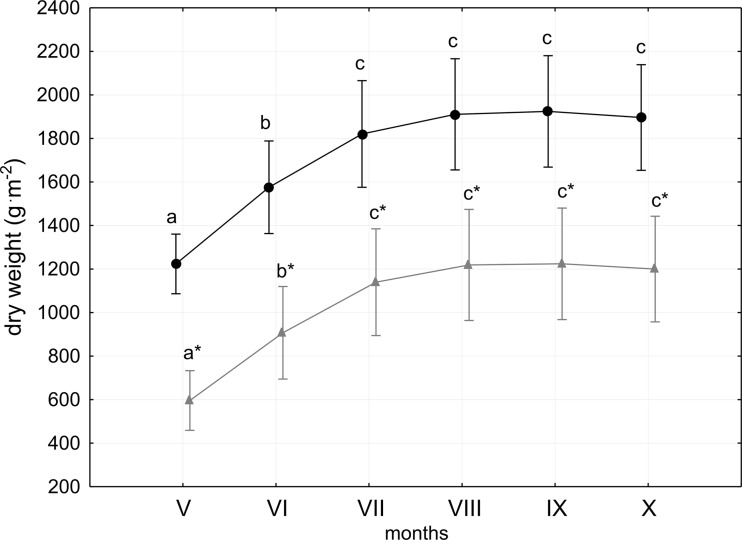
The month-to-month variability of charophyte dry weight at the 1 m (*black*) and 3 m depths (*grey*) in Lake Jasne. *Asterisks* (*) indicate significant (*P* < 0.05) depth-to-depth differences in particular months (*Roman digits*). *Different letters* indicate significant (*P* < 0.05) month-to-month differences. Midpoints and bars are the means ± 1 SD (*n* = 3 for each depth in each month)

The study sites (A, B, C) did not differ significantly with respect to the amounts of dry mass and calcium carbonates (ANOVA, *P* > 0.05). For each site, the values of both the dry weight and the calcium carbonates were significantly higher at 1 m than at 3 m (Table 1). Similar scatter over the range of data was found at both depths (Fig. 1). Although the dry weight at 1 m depth was twice as high as at 3 m (ANOVA, *P* < 0.05), the change in dry weight was similar over the investigation period. At both depths, the dry weight differed from month to month but the differences were significant for the first 3 months only. The lowest dry weight was observed in May (530 g m^−2^ at 3 m and 1324 g m^−2^ at 1 m at sites B and A, respectively), followed by a considerable increase to the maximum in September (1348 g m^−2^ at 3 m and 2089 g m^−2^ at 1 m, both at site A). The greatest increase in dry weight occurred between May and June (a 23 % increase at 1 m and 36 % increase at 3 m, both at site A). Between June and July, the increase was approximately one third less. The smallest increase in dry weight occurred between June and July and did not exceed 1 % at any site. From August to October, the dry weights decreased slightly. In October (at the end of the investigation period), the dry weight at both depths was similar to those noted in June (1321 g m^−2^ at 3 m and 2050 g m^−2^ at 1 m at sites B and A, respectively).

**Table 1:** The range of charophyte dry weight and precipitated CaCO_3_^−^ and the results of one-way ANOVA tests for the 1 and 3 m depths (*n* = 18 for each depth)

	Dry weight (g m^−2^)		CaCO_3_ (g m^−2^)	
	1 m	3 m	1 m	3 m
Minimum	1111.50	529.75	720.80	311.66
Maximum	2089.00	1348.75	1695.47	1105.00
Mean ± SD	1725.06 ± 293.44	1047.61 ± 258.06	1255.45 ± 277.92	779.64 ± 247.50
Scheffe’s test (P)	<0.001		<0.001

**Fig. 2:**
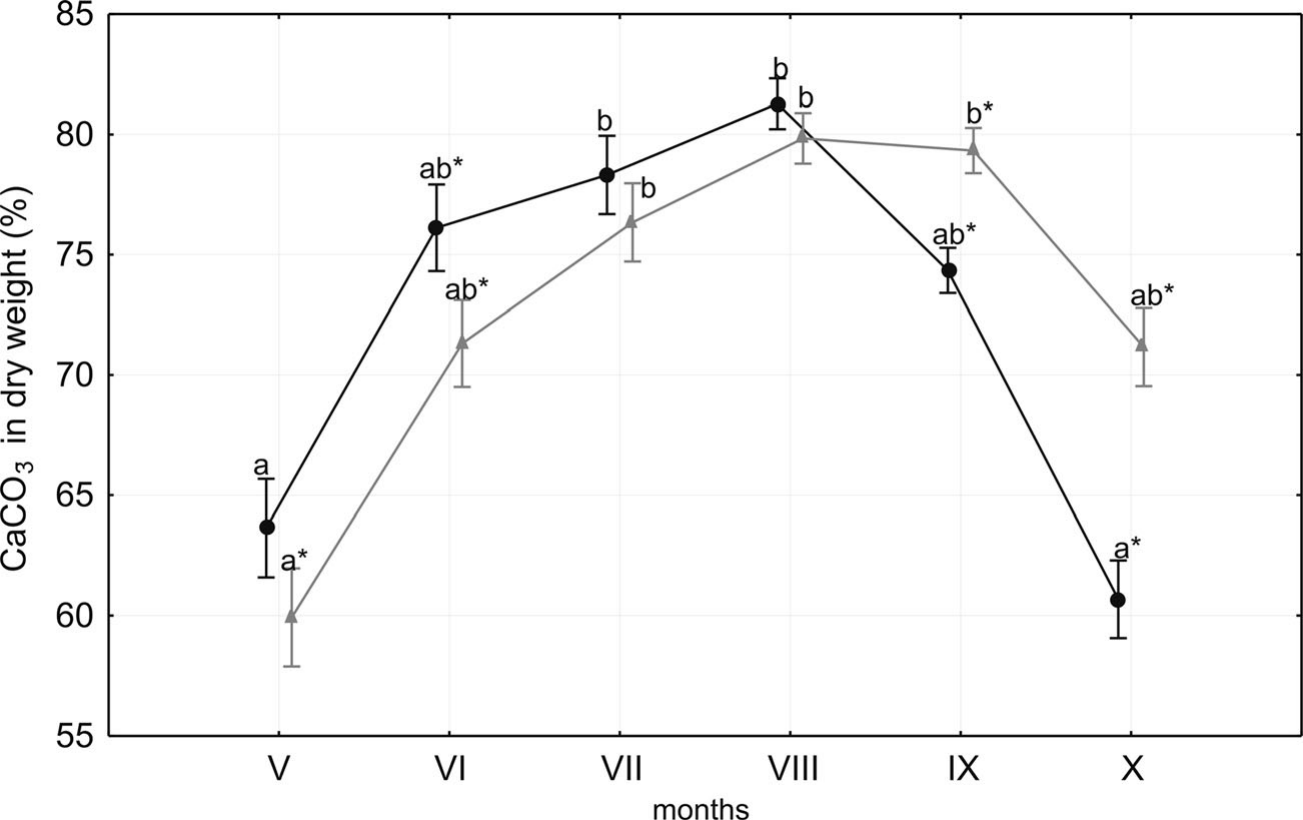
The month-to-month variability of calcium carbonate content in charophyte dry weight at the 1 m (*black*) and 3 m (*grey*) depths in Lake Jasne. *Asterisks* (*) indicate significant (*P* < 0.05) depth-to-depth differences in particular months (*Roman digits*). *Different letters* indicate significant (*P* < 0.05) month-to-month differences. Midpoints and bars are the means ± 1 SD (*n* = 3 for each depth in each month)

There were no significant differences (ANOVA, *P* > 0.05) in the calcium carbonate content of the dry weight between 1 and 3 m, although the month-to-month variation differed for each depth (Fig. 2). At the beginning of the growing season, calcium carbonate content was low at both depths (58.8 % at 1 m and 55.6 % at 3 m at sites A and C, respectively). As with the biomass, the calcium carbonate content increased considerably to a maximum value in August (82.3 % at 3 m and 81.8 % at 1 m at sites A and C, respectively). From May to August, the carbonate content in dry weight varied from 1.4 to 4.8 % higher at 1 m than at 3 m. In September, the carbonate content started to decrease. The decline was more rapid at 1 m, falling below the values recorded at 3 m. In October, the carbonate contents at 1 m were the lowest observed over the investigation period.

During the investigation period, calcium carbonate content was strongly correlated (*r* > 0.75, *P* < 0.05) with the dry mass at both 1 and 3 m.

### Variability in water properties

The analyses of physical-chemical parameters demonstrated spatial homogeneity of water at the study sites, which was reflected in low variability of the parameters (Table 2). No statistical differences (ANOVA, *P* > 0.05) were found among the study sites (A, B or C). All the parameters, except TP, varied significantly (ANOVA, *P* < 0.05) between months at both sampling depths throughout the study period. The only statistically significant differences between 1 and 3 m were found for PAR. At both depths, a similar pattern of PAR variability was observed; however, at the 1 m depth, PAR was distinctly higher for the entire investigation period (Table 2).

**Table 2:** The variability of the physical-chemical properties of water in the study sites at 1 and 3 m depths (*n* = 18 for each depth)

	Depth	Minimum	Maximum	Mean ± SD
Temperature (°C)	1 m	12.70	24.00	19.75 ± 3.59
	3 m	12.80	23.50	19.01 ± 3.40
O_2_ (mg L^−1^)	1 m	7.10	9.10	8.07 ± 0.68
	3 m	7.20	8.80	8.03 ± 0.51
pH	1 m	7.50	8.80	8.29 ± 0.43
	3 m	7.50	8.70	8.18 ± 0.40
Conductivity (μS cm^−1^)	1 m	238.00	324.00	258.89 ± 29.46
	3 m	239.00	315.00	259.28 ± 26.60
PAR (%)	1 m	70.49	89.16	78.09 ± 5.11*
	3 m	14.08	39.88	28.10 ± 7.60*
Alkalinity (mval L^−1^)	1 m	1.60	1.80	1.69 ± 0.06
	3 m	1.60	1.90	1.72 ± 0.08
Colour (mg Pt L^−1^)	1 m	5.00	12.00	8.89 ± 2.54
	3 m	6.00	12.00	9.00 ± 2.52
TP (mg L^−1^)	1 m	0.003	0.056	0.025 ± 0.018
	3 m	< 0.001	0.059	0.024 ± 0.020
TN (mg L^−1^)	1 m	1.05	1.72	1.35 ± 0.18
	3 m	1.17	2.23	1.41 ± 0.27
Ca^2+^ (mg L^−1^)	1 m	34.30	51.70	46.37 ± 4.99
	3 m	31.40	52.58	45.97 ± 5.48
Mg^2+^ (mg L^−1^)	1 m	1.41	10.90	4.82 ± 3.20
	3 m	1.46	9.10	4.72 ± 2.32
hardness (dH)	1 m	7.17	8.42	7.61 ± 0.30
	3 m	7.06	8.30	7.50 ± 0.31

*Significance level of *P* < 0.05

**Fig. 3:**
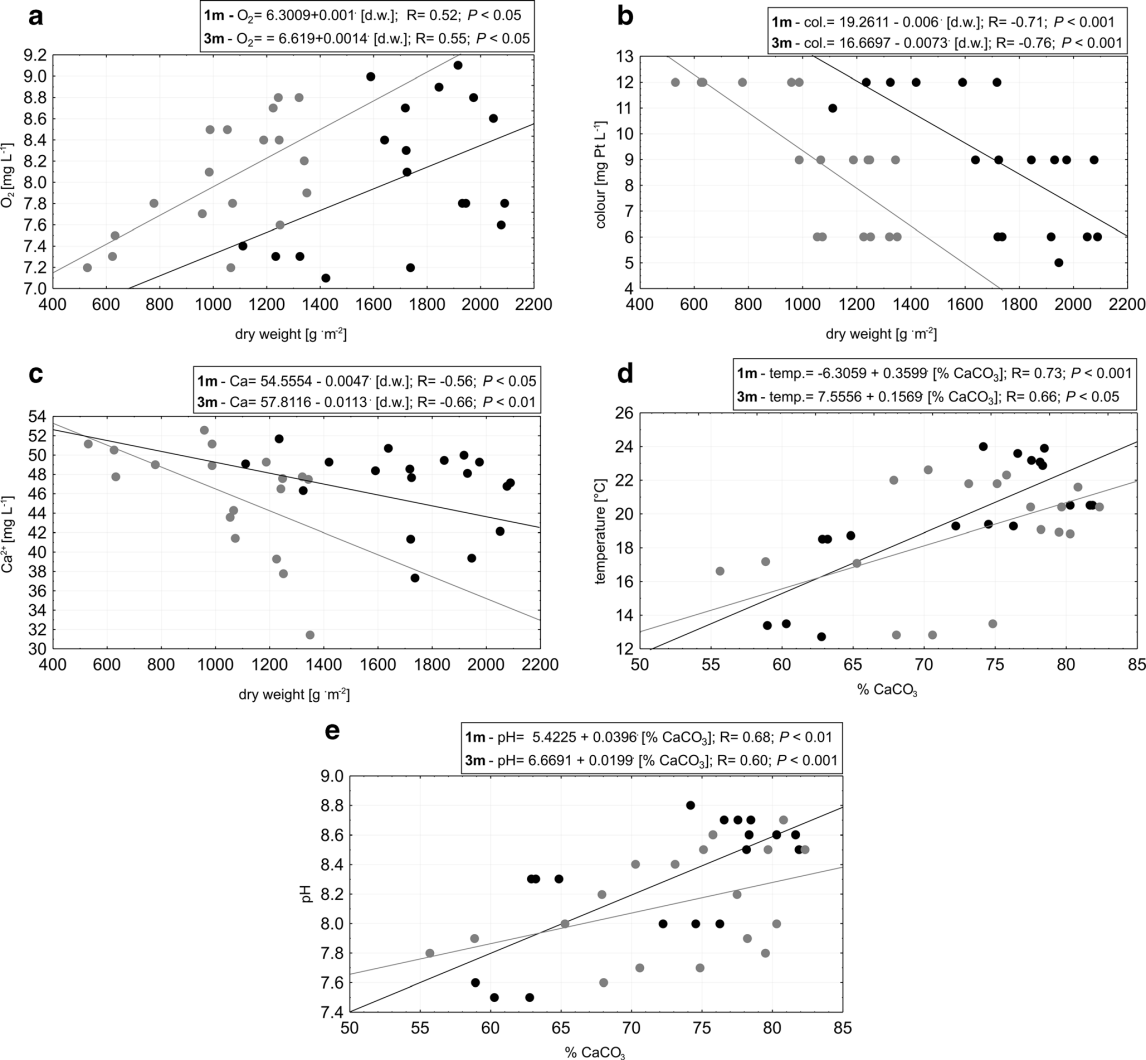

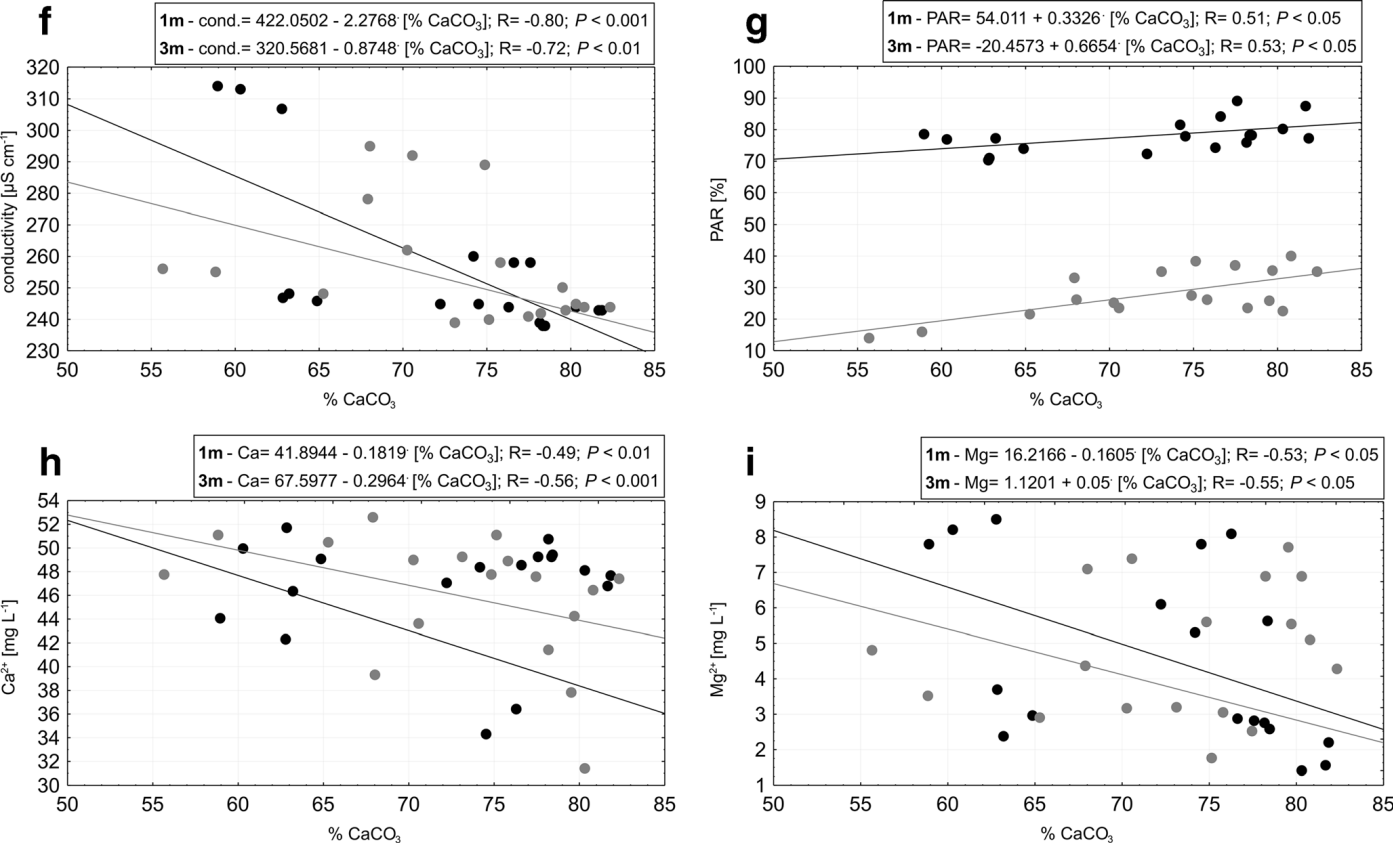
Correlations between the dry weight (**a**–**c**) and calcium carbonate content (**d**–**i**) and physicochemical properties of Lake Jasne water collected from the studied sites at 1 m (*black*) and 3 m (*grey*). Water properties: **a** oxygen, **b** colour, **c** Ca^2+^, **d** temperature, **e** pH, **f** PAR, **g** conductivity, **h** Mg^2+^, **i** Ca^2+^

While no spatial heterogeneity could be shown for the study sites, some of the physical-chemical parameters were significantly correlated (*P* < 0.05) with charophyte dry weight and calcium carbonate content (Fig. 3a–i). At both depths, the dry weight was positively correlated with oxygen concentration and negatively correlated with colour and calcium ions (Fig. 3a–c). Calcium carbonate content was positively correlated with temperature, pH and PAR but negatively correlated with conductivity, magnesium ions and calcium ions (Fig. 3d–i).

## Discussion

Lake Jasne is an example of a stable hard water lake, with environmental parameters enabling the development of extensive and diverse charophyte meadows, referred in the literature as *Chara*-lakes (Pukacz and Pełechaty 2013). Such a designation was also supported by the results of this study. Monospecific communities, occurring during the entire study period at all sampling sites, are indicators of a good ecological state that favours diverse vegetation and other biota (Van den Berg et al. 1998; Schwarz et al. 1999; Pukacz et al. 2013).

At all sites, charophytes produced considerable amounts of biomass. The mean and maximum values of dry mass exceeded 1500 and 2000 g m^−2^, respectively. These findings were similar to the values previously reported for Lake Jasne (2076 g m^−2^, Pukacz et al. 2014a) but were much higher than those noted by Królikowska (1997) from Lake Łuknajno (1200 g m^−2^). The values reported herein were also significantly higher than the mean charophyte dry weight of 279 g·m^−2^ calculated by Kufel and Kufel (2002) based on available published data and the average value of 623 g·m^−2^ given by Pełechaty et al. (2013). Our findings were also greater than the maximum and mean values presented by Blindow et al. (2002) for *C. tomentosa*, despite the fact that the pattern of month-to-month variability in the biomass of this species was similar to that of *C. polyacantha* in Lake Jasne. It must be stressed, however, that it is difficult to compare the results of this study to those previously published due to differences in methods, sites and species. For example, here, the plants were cut manually at the base of the plant, whereas in the other studies, different samplers (e.g. a Bernatowicz rake sampler or plexiglass core) of different sampling sizes were used. In addition, Lake Jasne is much smaller than the other lakes from which charophyte dry weights have been noted.

As summarized by Kufel and Kufel (2002), the dry weight per unit of the overgrown area may vary inter- and intra-specifically. Nevertheless, in general, the large charophytes (e.g. *C. polyacantha* and *C. tomentosa*) produce more dry weight than do the small species (e.g. *C. aspera* and *C. contraria*). However, this trend in growth depends on individual morphological features rather than on the length of the thallus or the community structure. According to Pukacz et al. (2014b), the individuals of *C. rudis* can produce more than twice as much dry weight compared with individuals of *C. polyacantha*. However, due to its specific morphology (long, branchy and very spiny), *C. polyacantha* forms extremely dense and uniform meadows within Lake Jasne, which results in remarkable biomass production per square metre (including calcium carbonate encrustation).

Our study revealed similar growth rates at 1 and 3 m despite significant differences in dry weight between the two depths. This finding is not entirely consistent with previous results from Lake Wigry (Pełechaty et al. 2013), where the greatest mean dry weight was noted at the 2 m depth. It should be noted, however, that monospecific charophyte meadows occurred only at a depth of 2 m in Lake Wigry, while mosses and angiosperms co-occurred with charophytes at the other two sampling depths (1 and 3 m).

Over 60 % of the lake bottom was overgrown by charophyte meadows (Pukacz and Pełechaty 2013; Pukacz et al. 2014a). It should also be emphasized that the dense structure of the charophyte meadows in Lake Jasne has remained stable for many years (10 years of observation data). Such a considerable amount of biomass is of great importance for the lake ecosystem, stabilizing the trophic status and stimulating clear water conditions (e.g. Scheffer 1998; Van Donk and van de Bund 2002; Blindow et al. 2002).

Currently, special attention is being paid to charophytes as they are highly efficient for nutrient trapping (Kufel and Kufel 2002). According to Kufel et al. (2013), the mean TP concentrations in the dry weight of different charophyte species range from 0.88 to 1.52 mg P g^−1^. The mean dry weights observed here were 1725 and 1321 g m^−2^, at 1 and 3 m, respectively; assuming 1.15 mg P·g^−1^ as the average TP value and depending on the time of year, the concentration of TP trapped in Lake Jasne can be calculated as ranging from 1.28 to 2.40 g m^−2^ (1.98 g m^−2^ on average) at 1 m and between 0.61 and 1.55 g m^−2^ (1.2 g m^−2^ on average) at 3 m. The values given by Królikowska (1997) for different charophyte species in Lake Łuknajno ranged from 0.10 to 0.43 P g m^−2^. However, in this case, the differences most probably resulted from the much greater charophyte dry weight noted in Lake Jasne.

We can assume that a significant proportion of phosphorus will be accumulated in an insoluble form as carbonates and be bound for a long time in the lake sediments (Schneider et al. 2015). Using the values given by Kufel et al. (2013), the fraction of calcium-bound phosphorus (HCl-SRP) may constitute, on average, 20.6 % of TP in the dry weight. Thus, we can assume that up to 0.50 g m^−2^ (calculated for the maximum dry weight) of phosphorus can be stored in the sediment.

Even with the partial decomposition of charophytes, which, based on our results constitutes less than 30 % (the difference between autumn and spring values) of the total biomass, the majority of lake nutrients will be immobilized for a long time in the charophyte biomass. Charophytes formed dense meadows (100 % bottom cover and ca. 30 cm thick) at all sites at the beginning of the study (1 month after ice melt), suggesting that the plants must have overwintered in Lake Jasne. This finding is in accordance with our earlier studies (Pełechata et al. 2015) and with underwater observations made during the winter season of 2011/2012. Samples taken at the 1 m depth at site B at the beginning of February 2012, under a 3-week ice cover, were approximately 1100 g m^−2^, which was similar to the minimum values from May 2010. Other authors have also suggested that charophytes overwinter (e.g. Pereyra-Ramos 1981; Blindow 1992a), which may be crucial for the functioning of the entire charophyte-dominated ecosystem. For example, we might expect that the accumulated nutrients remain stored in charophytes until the next growing season (Kufel and Kufel 2002). In addition, overwintering plants have an advantage over phytoplankton in competing for light and nutrients, which, among other mechanisms of intra-biocenotic interplay, makes such plants more effective in clear water maintenance (van den Berg et al. 1998). Further, during a severe winter season, charophyte meadows are an important niche for many animals, such as zooplankton (Scheffer 1999) and macroinvertebrates (Van den Berg et al. 1997; Blindow et al. 2014).

There is no data on the nutrient supply of Lake Jasne from the drainage basin to further characterize the real role of charophytes in nutrient trapping. Nonetheless, based on existing information and 10 years of observations, we can conclude that the nutrient concentrations remain stable in this ecosystem. The drainage basin is dominated by forests, and there is little anthropogenic pressure on the lake. There was also very low temporal and spatial variability of TN and TP in ambient water during this investigation.

However, little is known about the uptake of mineral substances by submerged vegetation, particularly in natural environments. However, based on the suggestions of Carignan (1982), we conclude that in mesotrophic Lake Jasne, charophytes uptake most of the phosphorus from the sediment.

Charophyte biomass and calcite precipitation depended on the depth, which is a consequence of light availability (Pełechaty et al. 2013 and references therein), as evidenced by the significant differences in biomass production between 1 and 3 m, although the dynamics of dry weight and calcium carbonate content were very similar at both depths. This dependence was confirmed by the significant differences in PAR between the sites at 1 and 3 m and was particularly evident at the beginning of the growing season (May–July), when the calcium carbonate content values were higher at more illuminated, shallow sites as a result of photosynthetic activity (Fig. 2). In contrast, biomass was greater at deeper sites in autumn (September–October). This difference in biomass, however, was probably a consequence of mechanical damage caused by waves and grazing by water birds (Kotta et al. 2004; Schmeider et al. 2006). The observations over preceding years suggest that Lake Jasne is a standing point for bean geese and swan in the autumn (September to October).

No site-to-site differences in the physical-chemical parameters indicate that there is substantial habitat homogeneity of the phytolittoral in Lake Jasne. The similar results were reported from earlier studies of the same water parameters from charophyte meadows in this lake, performed between April and September 2010 at a depth of 1.5 m (Pukacz et al. 2014a). The frequent mixing of epilimnetic water, as well as the influence of extensive charophyte meadows, may lead to this homogeneity.

The significant correlations of dry weight and calcium carbonate content with most of the physical-chemical parameters suggest a mutual relationship between charophyte biomass and water properties. In our opinion, these results demonstrate that charophytes are not only sensitive bioindicators but they also have the ability to modify habitat conditions (Kufel and Kufel 2002; Schneider et al. 2015).

The differences in correlation coefficients for the 1 and 3 m depths suggest that the relationships between charophyte biomass and carbonate content with water characteristics are depth dependent. Moreover, dry weight and calcium carbonate content should be considered separately.

The correlation between the dry weight and oxygen indicates that charophytes, in addition to phytoplankton (Pełechata et al. 2015), are one of the crucial primary producers in Lake Jasne. Another effect of charophyte growth was a decrease in Ca^2+^, as evidenced by the negative correlation of Ca^2+^ with dry weight and calcium carbonate content. This relationship was documented in this lake in earlier studies (Pełechaty et al. 2015) and is a consequence of the assimilation of soluble calcium bicarbonates, which are used as a source of CO_2_ in photosynthesis (McConnaughey 1997). Insoluble calcium carbonate is precipitated as an encrustation, which explains the relationships between Ca^2+^ content in water and CaCO_3_ in encrustations. The rate of this process is largely dependent on PAR availability and temperature, as evidenced by the positive correlations of these parameters with calcium carbonate content.

The strong negative correlation between dry weight and colour can be explained by the decrease in suspended solids resulting from charophyte growth. The highest values of colour were observed in May and June, coincident with a large inflow of rainwater with leached humic substances. As charophyte biomass increased, water colour decreased and water clarity improved; this phenomenon has also been observed in freshwater ecosystems dominated by macrophyte vegetation (Scheffer 1999).

Even though no direct correlation was found between biomass production and PAR, biomass was lower throughout the entire study period at deeper and less illuminated sites compared to shallower sites. Despite the depth-related differences in underwater light, PAR values did not fall below 14 % of the surface irradiance; thus, PAR availability at every studied site was sufficient for undisturbed photosynthesis and related biomass production (Brown et al. 2000).

The precipitation of carbonates and pH values were interrelated. Over the investigation period, carbonate content in the charophyte dry weight coincided with pH values; an increase was observed in the first half of the vegetation season and a decrease occurred in autumn. The use of bicarbonates is among the crucial factors determining the annual fluctuations of carbon in a hard water lake, with meaningful consequence for primary production in the first half of the year and for matter decomposition in the second half (Wetzel 2001). Although this effect was not evidenced in significant correlations with alkalinity, it was reflected in the season-to-season changes in electrolytic conductivity. A decrease in conductivity, with increasing dry matter and CaCO_3_ content during the growing season, might have been a result of photosynthetically induced water decalcification. Because Lake Jasne is isolated from a land-based external solute supply, other sources of bicarbonate and carbonate ions are rather unlikely to be the main determinants for the electrolytic conductivity (Wetzel 2001). The covariance of pH values seems to corroborate this conclusion.

In the case of negative correlation with magnesium ions, there are two possible explanations. First, the magnesium concentration could vary according to the abovementioned mechanism described for calcium concentration. However, that would only be the case if aragonite was precipitated. Second, according to the previous study in Lake Jasne (Pukacz et al. 2014a), favourable conditions for calcite precipitation occurred; thus, the correlation may be the effect of the inhibiting role of magnesium ions in the process of calcification, as suggested by Siong and Asaeda (2009).

Although our results are for one lake only, they have broad significance because of the widespread distribution of lakes with large littoral areas covered by charophytes. Our findings are relevant for both modern ecological and palaeolimnological studies. However, in our opinion, the results herein must be considered carefully because the data were gathered in situ; thus, many other factors could affect both the variability of charophyte biomass and water properties. A detailed study including a wider set of parameters (including phytoplankton and zooplankton structure) and analyses of lacustrine sediment (particularly in the context of nutrient cycling) would be desirable.

## Conclusions

In all the studied sites, there were favourable conditions for the growth of charophytes and precipitation of calcium carbonate. It was reflected in great amounts of biomass, exceeding 2000 g m^−2^ of d.w. at the peak of the growing season, out of which calcium carbonate constituted over 80 %. These values are the highest known so far from literature and emphasize the high efficiency of charophytes in CaCO_3_ precipitation and sedimentation.

With regard to extensive charophyte meadows that can overwinter, the results seem to indicate a great habitat-engineering role of charophytes in lakes. It was stressed by correlations between the biomass of charophytes and physical-chemical properties of water. However, some of the correlations (e.g. with PAR), as well as the differentiation of biomass (including carbonate encrustation), show that charophyte productivity is habitat dependent. Consequently, it can be concluded that the amount of precipitated carbonates may reflect changing habitat conditions. This can apply both in modern ecological studies and palaeolimnological studies. It is necessary, however, to recognize in greater detail the impact of charophyte biomass on lacustrine sediments (particularly in the context of nutrient cycling), which will also be considered in our further study.
